# The complete chloroplast genome of an Endangered species *Cymbidium Nanulum* (Orchidaceae)

**DOI:** 10.1080/23802359.2019.1638327

**Published:** 2019-07-13

**Authors:** Yong-Qiang Zhang, Ting-Zhang Li, Jie Huang, Gui-Zhen Chen, Guo-Qiang Zhang

**Affiliations:** a Key Laboratory of National Forestry and Grassland Administration for Orchid Conservation and Utilization, Shenzhen, China;; b Shenzhen Key Laboratory for Orchid Conservation and Utilization, The National Orchid Conservation Centre of China and The Orchid Conservation and Research Centre of Shenzhen, Shenzhen, China

**Keywords:** *Cymbidium nanulum*, chloroplast genome;*·*IUCN

## Abstract

*Cymbidium nanulum* Y.S.Wu & S.C.Chen is an IUCN Red listed Endangered species and distributes in South-Central China and Hainan. Here, we report the complete chloroplast (cp) genome sequence and the cp genome features of *C. nanulum.* The complete chloroplast (cp) genome sequence of *C. nanulum* was 149,776 bp in length. It presented a typical quadripartite structure including one large single-copy region (LSC, 85,392 bp), one small single-copy region (SSC, 14,210 bp), and two inverted repeat regions (IRs, 25,087 bp). The cp genome encoded 133 genes, of which 104 were unique genes (77 protein-coding genes, 23 tRNAs, and 4 rRNAs). The maximum-likelihood phylogenetic analysis showed that *C. nanulum* was closely related to other species of genus *Cymbidium.*

Since the genus *Cymbidium* was established by Swartz (1799, p. 6), some infrageneric treatments have been published, such as those by Schlechter (1924); Hunt (1970); Seth and Cribb (1984); Puy and Cribb (1988); Berg (2002); Liu et al. (2006); Chen et al. (2009). Currently, approximately 80 species are recognized within the genus. The genus belongs to the subtribe Cymbidiinae (Orchidaceae) and ranges from tropical and subtropical Asia south to Australia (Chen et al. 2009; Pridgeon et al. 2009). China is the distribution centre of *Cymbidium* with over 50 species, of which 19 endemic (Liu et al. 2006; Chen et al. 2009; Chen et al. 2019; Zhang et al. 2019). *Cymbidium* orchids are quite popular in flower markets due to their aesthetic appeal and ideal characteristics as a house plant.

So far, the complete chloroplast genome of *Cymbidium* have been reported, such as *C. lancifolium*, *C. kanran*, *C. ensifolium* (Shi et al. 2017). These studies may contribute to the study of species identification, germplasm diversity, genetic engineering (Lin et al. 2015). The complete chloroplast genome sequence of *C. nanulum* was assembled in this study.

Leaf samples of *C. nanulum* were obtained from the Orchid Conservation and Research Centre of Shenzhen and specimens were deposited in the National Orchid Conservation Center herbarium (NOCC; specimen code Z.J.Liu 2562). Total genomic DNA was extracted from fresh material using the modified CTAB procedure of Doyle and Doyle (1987). Sequenced on Illumina Hiseq 2500 platform (San Diego, CA, USA). Genome sequences were screened out and assembled with MITObim v1.8 (Hahn et al. 2013), which resulted in a complete circular sequence of 144,940 bp in length. The cp-genome was annotated with CpGAVAS (Liu et al. 2012).

The cp genome sequence of *C. nanulum* (GenBank accession MK820372) was 149,776 bp length and presented a typical quadripartite structure including one large single-copy region (LSC, 85,392 bp), one small single-copy region (SSC, 14,210 bp), and two inverted repeat regions (IRs, 25,087 bp). The cp genome encoded 133 genes, of which 104 were unique genes (77 protein-coding genes, 23 tRNAs and 4 rRNAs).

A molecular phylogenetic tree was constructed with the maximum-likelihood (ML) methods employing a data set of the complete cp genome sequences of 12 species from the genus *Cymbidium*. The ML analysis was performed using the CIPRES Science Gateway web server (RAxML-HPC2 on XSEDE 8.2.10) with 1000 bootstrap replicates and settings as described by Stamatakis et al. (2008). The result showed that they were all clustered together (Figure 1). The characterized chloroplast genome sequence of *C. nanulum* will be helpful for further study on the phylogenetic study, species identification, and genetic engineering.

**Figure 1.**
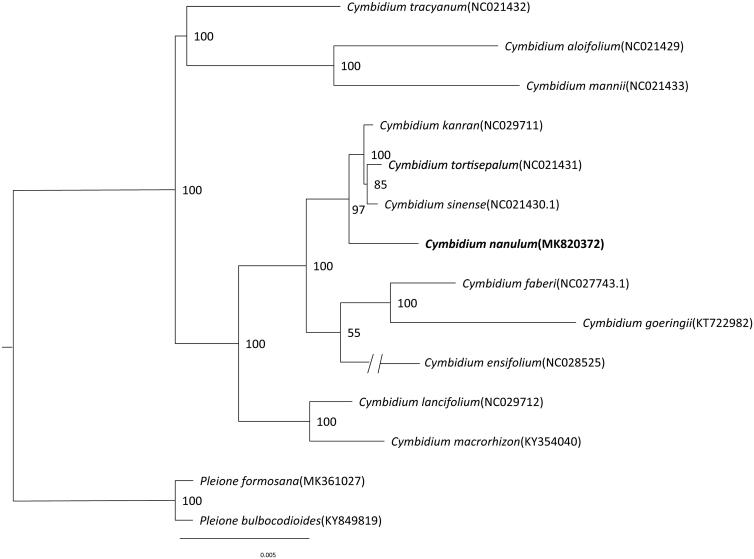
Phylogenetic position of *Cymbidium nanulum* inferred by maximum likelihood (ML) of complete cp genome. The bootstrap values are shown next to the nodes.
